# Insight into Evolution, Processing and Performance of Multi-length-scale Structures in Planar Heterojunction Perovskite Solar Cells

**DOI:** 10.1038/srep13657

**Published:** 2015-09-04

**Authors:** Yu-Ching Huang, Cheng-Si Tsao, Yi-Ju Cho, Kuan-Chen Chen, Kai-Ming Chiang, Sheng-Yi Hsiao, Chang-Wen Chen, Chun-Jen Su, U-Ser Jeng, Hao-Wu Lin

**Affiliations:** 1 Institute of Nuclear Energy Research, Longtan, Taoyuan 32546, Taiwan; 2 Department of Materials Science and Engineering, National Tsing Hua University, Hsinchu 30013, Taiwan; 3 National Synchrotron Radiation Research Center, Hsinchu 30076, Taiwan

## Abstract

The structural characterization correlated to the processing control of hierarchical structure of planar heterojunction perovskite layer is still incomplete due to the limitations of conventional microscopy and X-ray diffraction. This present study performed the simultaneously grazing-incidence small-angle scattering and wide-angle scattering (GISAXS/GIWAXS) techniques to quantitatively probe the hierarchical structure of the planar heterojunction perovskite solar cells. The result is complementary to the currently microscopic study. Correlation between the crystallization behavior, crystal orientation, nano- and meso-scale internal structure and surface morphology of perovskite film as functions of various processing control parameters is reported for the first time. The structural transition from the fractal pore network to the surface fractal can be tuned by the chloride percentage. The GISAXS/GIWAXS measurement provides the comprehensive understanding of concurrent evolution of the film morphology and crystallization correlated to the high performance. The result can provide the insight into formation mechanism and rational synthesis design.

Organometal halide perovskite solar cells have recently attracted considerable attention due to the advantages of high performance, low cost, solution-processablity, light weight and flexibility^1^,^2^,^3^,^4^,^5^,^6^,^7^,^8^,^9^,^10^,^11^. The perovskite solar cells demonstrate the exciting progress with power conversion efficiency (PCE) up to ~19.3%^2^,^4^,^6^,^9^,^10^,^11^. The perovskite solar cells develop toward commercialization considering the easy fabrication and simplified device architecture. The evolution of optimum cell structure is directed to planar heterojunction with perovskite thin film sandwiched between p- and n- type charge collecting layers from meso-superstructured solar cell (MSSC) with metal-oxide mesoporous scaffold deposited by the perovskite absorber^11^,^12^,^13^,^14^. The high-performance planar n-i-p heterojunction perovskite solar cells can be fabricated by the different methods: (1) sequential vacuum deposition^2^,^12^, (2) vacuum co-deposition,4 (3) one-step solution-processed deposition (two precursor solutions are mixed and then coated on substrates followed by thermal treatment)^5^,^7^,^15^,^16^, (4) two-step solution-processed deposition, or called sequential deposition, (a PbI_2_ or PbCl_2_ layer is first deposited by spin-coating and then sequentially react with CH_3_NH_3_I solution or vapor for transformation into CH_3_NH_3_PbI_3_ or CH_3_NH_3_PbI_3−x_Cl_x_ perovskite structure)^2^,^6^. Perovskite film structure, usually characterized by the uniformity, coverage and pin-hole (or gap) between grains, is the critical role largely affecting the PCE^3^,^17^,^18^.

Several reports pointed out that the uniform and full-coverage perovskite film comprised of large crystal grains can significantly enhance PCE and improve stability^19^,^20^. The above structural characteristics of perovskite film (/and grain) can be tuned by synthesis parameters, such as solution concentration, deposition way, solvent-drying control and thermal treatment^3^,^21^,^22^. These structural structures are closely related to the exciton diffusion, charge carrier disassociation and transport to the electrodes. To date, the characterization of these structures is focused on the surface morphology probed by scanning electronic microscopy (SEM) and atomic force microscopy (AFM). Generally, the quantitative characterization of internal structure of perovskite film is still lack. On the other hand, it is reported that the crystalline characteristics of perovskite layer play important roles affecting the cell performance^12^,^19^. However, the crystalline structure of the perovskite film was usually studied by conventional X-ray diffraction (XRD). It is pointed out that XRD provides the limited information due to the existence of prefer-orientation of perovskite grains^23^. In contrast, grazing incidence wide-angle X-ray scattering (GIWAXS) using two-dimensional (2D) detector can give the sufficient crystalline information, including the orientation of all perovskite grains inside film^22^,^23^. Very few studies reported the comprehensive characterization of the structures at the different scale levels in the bulk perovskite film from the oriented crystalline structure to the internal and surface morphology of perovskite film.

The simultaneously grazing-incidence small-angle scattering and wide-angle scattering (GISAXS/GIWAXS) techniques are an effective tool to quantitatively probe the hierarchical structure of phase-separated bulk heterojunction structure in the polymer solar cells over the current structural observations^24^,^25^,^26^,^27^,^28^,^29^,^30^. This present study performed the simultaneously synchrotron GISAXS/GIWAXS measurement to quantitatively characterize the multi-length-scale structures of the bulk CH_3_NH_3_PbI_3−x_Cl_x_ perovskite films prepared by different control conditions under (1) sequential vacuum deposition^12^ and (2) one-step solution-processed deposition. Correlation between the crystallization behavior (in terms of crystallinity, decomposition and orientation of grains), nano- and meso-scale internal morphology (i.e., porous structure), and surface morphology of perovskite film as functions of various parameters of growth control is reported for the first time. The result can provide the insight into formation mechanism and rational synthesis design. In one-step solution-processed deposition method, the evolution of hierarchical pore-network structure inside the perovskite film into a dense (no internal pore) grain structure with fractal surface can be tuned by preparation parameters. In sequential vacuum deposition method, the quantitative GISAXS analysis reveals how fractal surface of densely aggregated grains into film evolves by tuning the substrate temperature. The corresponding GIWAXS patterns demonstrate the variation of orientation of crystalline grains and phase transformation. The relationship among photovoltaic performance, multi-length-scale structures of film and crystalline characteristics of perovskite grains is discussed herein. The new information firstly obtained by GISAXS/GIWAXS study provides the comprehensive understanding of how the multiple-length-scale structures evolve under the different fabrication control and formational mechanism for the planar heterojunction perovskite solar cells.

## Results

For the CH_3_NH_3_PbI_3−x_Cl_x_ perovskite films prepared by the sequential vacuum deposition method (the details is described elsewhere)^12^, the critical parameter controlling the PCE and film structure is the substrate temperature for the vapor deposition of CH_3_NH_3_I on the PbCl_2_ film. The CH_3_NH_3_PbI_3−x_Cl_x_ perovskite films prepared at the substrate temperatures of 65, 75 and 85 °C, respectively, were investigated by the simultaneously synchrotron GISAXS/GIWAXS measurement. For the CH_3_NH_3_PbI_3−x_Cl_x_ perovskite films prepared by the one-step solution-processed deposition method (the details is described in the Experimental section), the key fabrication parameter is the percentage of chloride (PbCl_2_) contained in the precursor solution (PbCl_2_ + PbI_2_ + CH_3_NH_3_I). We used the GISAXS/GIWAXS to investigate the CH_3_NH_3_PbI_3−x_Cl_x_ perovskite films prepared with 0, 10, 20 and 40% of chloride (Cl/(Cl + I); at.%), respectively. Finally, the CH_3_NH_3_PbI_3−x_Cl_x_ perovskite films prepared with the optimum percentage (20%) of chloride and annealed at 100 °C for 1, 5, 10, 15 and 60 min, respectively, were investigated by the GISAXS/GIWAXS measurement to understand the effect of the annealing time on the structural evolution. For investigating their photovoltaic performance, the planar heterojunction perovskite solar cell based on the above films were fabricated with the architecture of ITO glass/PEDOT:PSS/perovskite/PC61BM/Bphen/Ca/Ag. For avoiding the interference effect of multiple scattering due to the grazing-incidence geometry^31^,^32^, the typical GISAXS profiles are represented by the in-plane GISAXS profiles (cut from the horizontal Yoneda peak^32^ in the 2D GISAXS pattern). These 1D GISAXS profiles were reduced from the 2D GISAXS patterns along the in-plane direction (parallel to the substrate or film surface) and are expressed as function of scattering vector *Q*.

**Figure 1:**
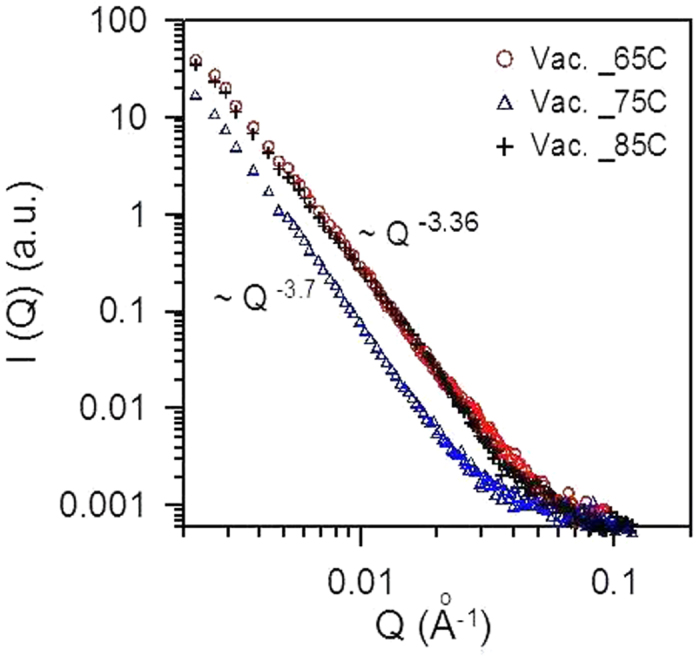
GISAXS profiles of the vacuum-deposited CH_3_NH_3_PbI_3−x_Cl_x_ films prepared at the substrate temperatures of 65, 75 and 85 °C, respectively.

**Figure 2:**
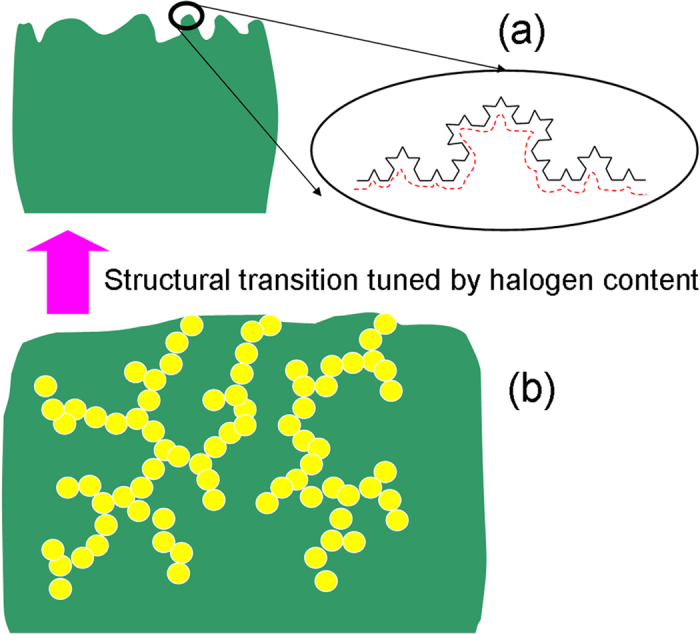
Schematic representation of (a) the dense perovskite grain with surface morphology of self-similarity at different scales indicated by black curve (red dashed curve represents the morphology of lower surface fractal dimension *D*_*s*_) and (b) the fractal pore network in the perovskite grain (yellow sphere represents the primary pore).

Figure 1 shows the GISAXS profiles, *I(Q)*, of the vacuum-deposited CH_3_NH_3_PbI_3−x_Cl_x_ films at the substrate temperatures of 65, 75 and 85 °C, respectively. The GISAXS profiles show the behavior of power-law scattering with the characteristic of surface fractal (*I(Q)* *∝* *Q*^*−α*^; 3 ≤ *α* ≤ 4)^33^,^34^. The exponent *α* is related to the surface fractal dimension *D*_*s*_ by *D*_*s*_ = *6-α*. The surface fractal reveals that film surface has the morphology of self-similarity at different scales starting from nanometers, schematically showing in Fig. 2. The SAXS technique is particularly useful for characterizing fractal aggregation structures/or fractal morphology using the power-law scattering behavior over a wide Q range, which is reported by a large number of papers^33^,^34^,^35^,^36^. The general microscopic tools (SEM, AFM, TEM etc.) are very difficult to identify the fractal morphology. Therefore, the SAXS characterization of the fractal system is complementary to TEM observation. The local morphology observed through TEM may provide a very rough evidence of a fractal object at a certain length scale. The previous study^37^ reported the fractal morphology in the different perovskite solar cells studied by GISAXS technique, which was correlated to the SEM (for macroscale morphology of large grain) and TEM (nanoscale fractal pore network). This power-law scattering also implies that the internal structure of film comprised of perovskite grains is dense (solid-state) or there is almost no micropore inside the film (or no mesopore between grains). The GISAXS profiles (Fig. 1) shows that the fractal surface morphologies formed at 85 and 65 °C are very similar. In contrast, the surface morphology formed at 75 °C shows a larger scale, evidenced by the power-law scattering at the smaller *Q* region. This result is consistent with the SEM observation for the films prepared by the same procedure previously reported^12^. The SEM images showed that all films have the full surface coverage of μm-scale grains. The corresponding AFM measurement shows the *R*_*rms*_ values of ~23 nm. The previous study^12^ reported that the CH_3_NH_3_PbI_3−x_Cl_x_ film prepared at 75 °C has much higher PCE (~15%) than those prepared at the other temperatures (6.1% and 4.5% for the subtract temperatures of 65 and 85 °C, respectively). It can be attributed to that the film prepared at 75 °C has the highest CH_3_NH_3_PbI_3_ crystallinity, evidenced by the simple one-dimensional (1D) XRD patterns previously reported^12^. It seems that the grain morphology is closely related to its crystalline structure and quality.

**Figure 3:**
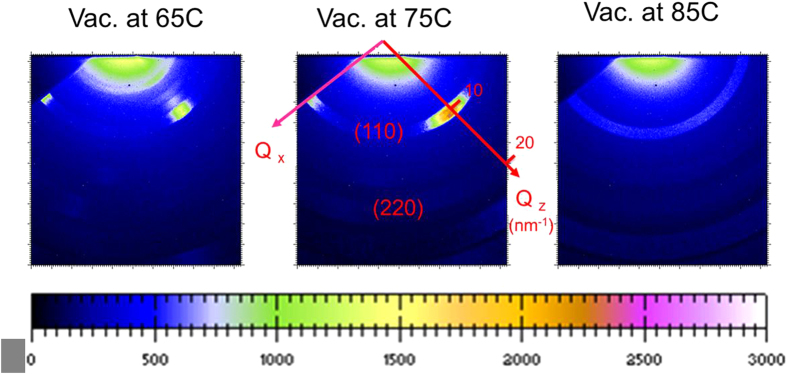
Two dimensional GIWAXS patterns corresponding to the vacuum-deposited CH_3_NH_3_PbI_3−x_Cl_x_ films prepared at the substrate temperatures of 65, 75 and 85 °C, respectively.

In this present study, all 2D GIWAXS patterns (Fig. 3) show the diffraction rings corresponding to the (110) and (220) planes at *Q* = 10 and 20 nm^−1^, respectively, being consistent with the CH_3_NH_3_PbI_3−x_Cl_x_ crystal structure pattern^22^,^23^. As shown in Fig. 3, the crystallites (/grains) with the (110) plane oriented (normal) to the out-of-plane direction dominates for all films^25^. The out-of-plane direction is perpendicular to the substrate or film surface (defined as *Q*_*z*_ direction marked in the 2D GIWAXS pattern). These oriented crystallites are indicated by the clear diffraction spot in the *Q*_*z*_ direction. The perovskite films prepared at 65 and 75 °C also show that a significant fraction of crystallites having the (110) plane oriented to the in-plane direction^25^, as indicated by the diffraction spot in the in-plane direction. The in-plane direction is parallel to the substrate or film surface (defined as the *Q*_*x*_ direction also marked in Fig. 3). The relative crystallinity can be approximately represented by the azimuthally averaged intensity of diffraction (110) ring in the 2D GIWAXS pattern. The perovskite film prepared at 75 °C has the highest crystallinity and the largest amount of dominated crystallites with (110) plane oriented to the out-of-plane direction, evidenced by diffraction (110) spot. The new information revealed in this GIWAXS study is as follows: (1) For the film prepared at 65 °C, a fraction of CH_3_NH_3_PbI_3−x_Cl_x_ crystallites rapidly decomposed into PbI_2_ crystallites^23^, as evidenced by the appearance of diffraction spot at *Q* = 9 nm^−1^ in the out-of-plane direction. (2) For the film prepared at 85 °C, a significant reduction of out-of-plane-oriented crystallites was accompanied by the formation of randomly-oriented crystallites, indicated by the isotropic distribution of diffraction ring in the 2D GIWAXS pattern (Fig. 3). The result suggests that the key parameter controlling or tuning the decomposition of vacuum-deposited CH_3_NH_3_PbI_3−x_Cl_x_ crystallites into PbI_2_ is the substrate temperature. The literature^20^ pointed out that the existence of appropriate amount of PbI_2_ can protect from the interaction with oxygen and water. According to the azimuthally averaged intensities of GIWAXS patterns, the relative crystallinity of CH_3_NH_3_PbI_3−x_Cl_x_ crystallites prepared at 85 °C is larger than that prepared at 65 °C. However, the PCE of the former (4.5%) is less than that of the latter (6.1%). It can be speculated that the grain boundary (GB) of all randomly-oriented crystallites forms an isotropic distribution of network. In contrast, the GB network formed by the dominated crystallites with the out-of-plane orientation (normal to the film surface) has an alignment structure which is more favorable to the transport of charge carrier. A recent literature reports that GB plays a beneficial role in collecting charge carriers efficiently^38^. It reveals that the influence of crystal orientation on the PCE is larger than that of crystallinity, showing the importance of the crystallites with the out-of-plane orientation. This case becomes critical for the films without high crystallinity. The high substrate temperature is the control factor changing the crystal orientation from the domination of the out-of-plane orientation to the random or isotropic orientation. The 2D GIWAXS patterns investigated here do not consider the geometric effect caused by the planar detector^25^, leading to large uncertainty. Therefore, we focus on the qualitative interpretation and relative comparison.

**Figure 4:**
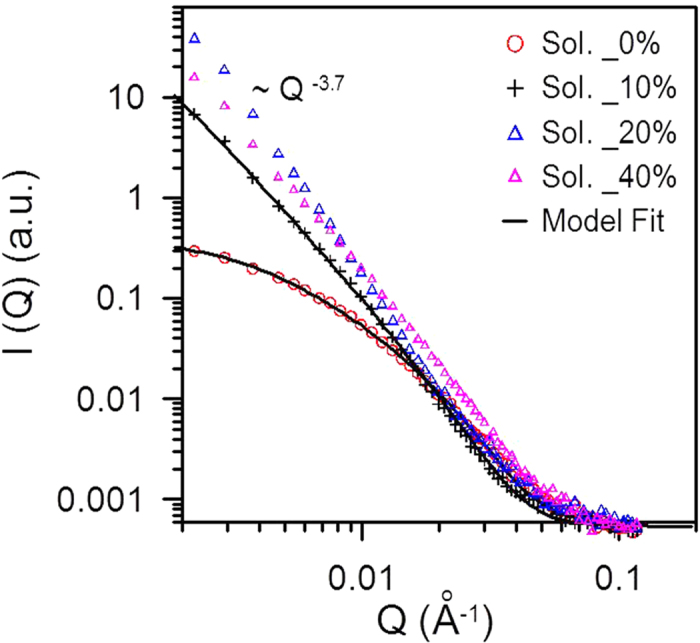
GISAXS profiles of the one-step solution-processed CH_3_NH_3_PbI_3−x_Cl_x_ films prepared with 0, 10, 20 and 40% of chloride, respectively.

Figure 4 shows the GISAXS profiles of the one-step solution-processed CH_3_NH_3_PbI_3−x_Cl_x_ films prepared with 0, 10, 20 and 40% of chloride, respectively. The GISAXS profiles prepared with 0 and 10% of chloride show the behavior of power-law scattering (*I(Q)* *∝* *Q*^*−α*^; 1 ≤ *α* ≤ 3) in the middle *Q* range (0.007 ~ 0.03 Å^−1^). Here, the exponent values, *α*, reveal the typical characteristic of mass fractal^33^,^34^,^35^. The only best fitting model needs to include the structure factor of fractal network comprised of the primary particles/or pores. The GISAXS is mainly contributed by the morphology of multi-length-scale fractal network structure formed from the aggregation of primary pores here. The GISAXS intensity profile can be expressed as^34^,^35^,^36^


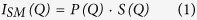


where *P(Q)* is the form factor of spherical pores as primary unit, and *S(Q)* is the structure factor, describing the interaction between primary pores in this fractal-like aggregation network system. In this aggregation system, the pores are inter-connected into the open pore channel network. *P(Q)* includes a pre-factor which is the product of pore volume fraction *φ* and the square of scattering length density contrast between pore and matrix. *S(Q)* is given by^34^,^35^,^36^


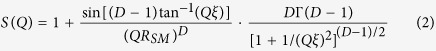


where *ξ* is the characteristic length of the fractal-like network domain (formed by the aggregation of the primary pores). *D* is the fractal dimension. The least-squares fitting calculation^39^ of equation (1) considers the polydispersity, *p*, of primary pores having a Schulz size distribution with mean radius *R*. Note that the fitted volume fraction, *φ*, (not discussed herein) is only a relative value and has large uncertainty because the absolute scattering contrast and the probed volume in the film cannot be measured. The domain size of the fractal network can be approximated by the Guinier radius (i.e., radius of gyration; *R*_*g*_* = [D(D + 1)/2]*^*1/2*^*ξ)*^34^. The fractal pore network in the perovskite grain is illustrated in Fig. 2.

**Figure 5:**
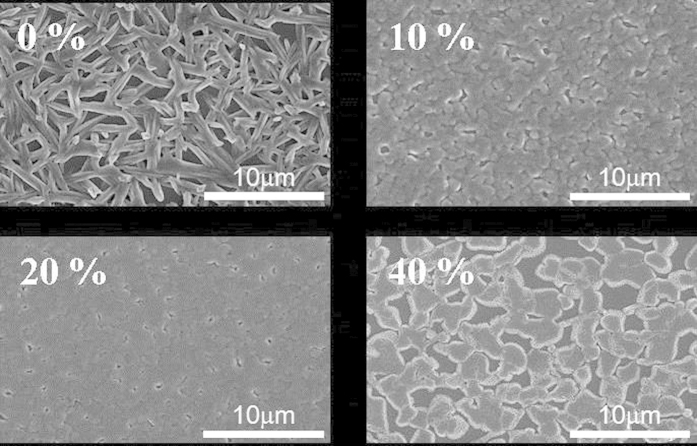
SEM images of the CH_3_NH_3_PbI_3−x_Cl_x_ films prepared with 0, 10, 20 and 40% of chloride, respectively.

Table 1 shows the structural parameters determined by the model fitting of Equation (1). Within the perovskite grains/or film prepared with 0% of chloride precursor (i.e., CH_3_NH_3_PbI_3_), there is an internal pore structure comprised of polydispersed primary pores of 3.3 nm in mean radius. The primary pores aggregate into a fractal network with fractal dimension of 1.8 and domain size (2*R*_*g*_) of ~56 nm. This result is very close to the GISAXS analysis of the two-step solution-processed CH_3_NH_3_PbI_3_ film prepared by an independent group^37^. The openness and local channel morphology of the fractal pore network were consistently observed along the 3D direction by the secondary ion mass spectrometry and high-resolution TEM studies, respectively^37^, for validating the SAXS analysis model. Interestingly, the GISAXS profile (Fig. 4) of the perovskite film prepared with 10% of chloride demonstrates a distinctive fractal pore network. The radius of primary pore, pore fractal dimension and network domain determined by the model analysis (Table 1) are significantly enhanced to 4.0 nm, 2.6 and 115 nm, respectively. Why the internal pore network evolves into a 2D-like and larger domain can be attributed to the morphological evolution of CH_3_NH_3_PbI_3−x_Cl_x_ perovskite film from the low-coverage aggregation of strip-like grains to the high-coverage aggregation of plane-like grains, as shown in the SEM observation (Fig. 5). Noticeably, when the chloride percentage is increased to 20%, an almost full coverage film is formed. The corresponding GISAXS profile (Fig. 4) shows a power-law scattering with the characteristic of surface fractal morphology (*I(Q)* *∝* *Q*^*−α*^; *α* = 3.7; *D*_*s*_ = 2.3) having a dense internal structure. Apparently, there is a structural transition from the internal pore network to surface fractal morphology. It is schematically shown in Fig. 2. It can be explained that the compact and high- density aggregation of perovskite grains forced the internal pores to collapse into a dense structure only with fractal morphology on the surface. Moreover, when the chloride percentage continues to increase to 40%, the aggregation of grains into film becomes over-coalescence and thus there appear a few holes between large grains (Fig. 5). The corresponding GISAXS profile shows the surface fractal morphology with fractal dimension α of ~3.0 (*D*_*s*_ = 3). The increase in surface fractal dimension reflects that the surface roughness becomes larger. The AFM measurement consistently shows that the *R*_*rms*_ values of the films prepared with 20 and 40% chloride precursor are 23.8 and 133 nm, respectively. The corresponding AFM images for the four perovskite films are shown in the supporting information (Figure S1). Compared to the microscopic observation, the GISAXS analysis can provide the insight into the 3D internal fractal pore structure and surface fractal morphology of CH_3_NH_3_PbI_3−x_Cl_x_ perovskite films at the nano- and meso-scale. Moreover, tuning the chloride content for the one-step processing to induce the structural transition from the internal pore network to the surface fractal morphology is reported for the first time. It provides the useful information to fabrication design and optimization.

**Figure 6:**
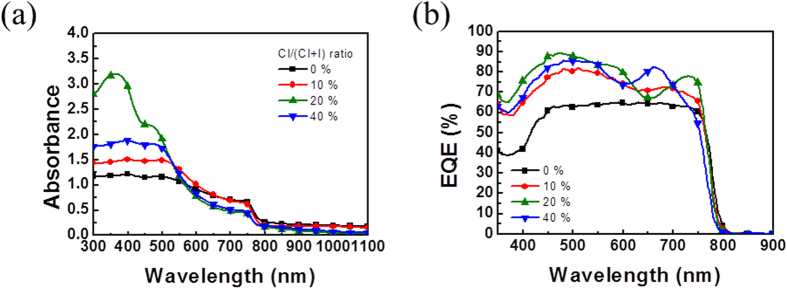
(**a**) The UV-vis light absorption spectra and (**b**) EQE spectra for the perovskite films as a function of chloride content.

**Figure 7:**
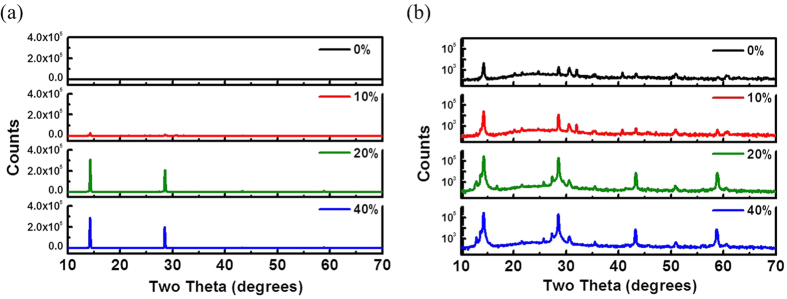
XRD patterns (a) in linear and (b) in logarithmic scale of the CH_3_NH_3_PbI_3−x_Cl_x_ films prepared with 0, 10, 20 and 40% of chloride, respectively.

**Figure 8:**
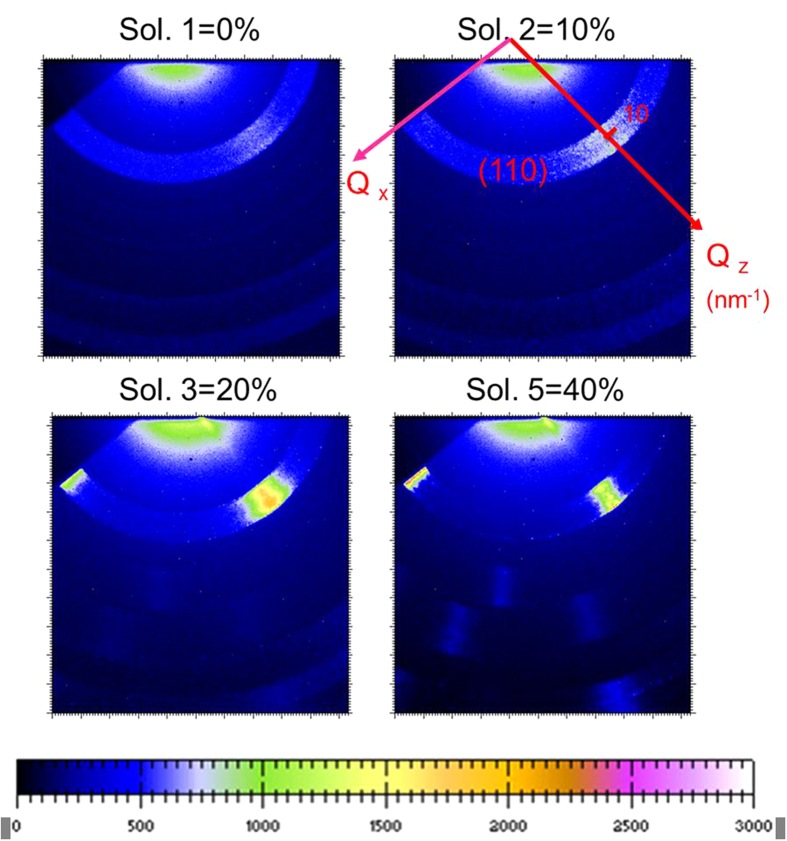
Two dimensional GIWAXS patterns of the one-step solution-processed CH_3_NH_3_PbI_3−x_Cl_x_ films prepared with 0, 10, 20 and 40% of chloride, respectively.

The film coverage of the perovskite layer prepared with 10% of chloride is much better than that of the layer prepared with 40% of chloride. The former also has an open internal pore network, which seems to be favorable to the dissociation and transport of charge However, The PCE (4.92%) of the former is much less than that (11.4%) of the latter. As shown in Fig. 6, the light absorption spectra and EQE spectra for the investigated perovskite films are consistent with the measured PCE values (in order, 20% > 40% > 10% > 0%, expressed by chloride ratio). The higher absorbance for the 20% Cl of sample is mainly due to the best surface coverage and almost pinhole free morphology of the perovskite thin film, which is revealed in the SEM image as shown in Fig. 5. Other samples exhibit much higher pin-hole (void) densities which largely decrease the absorbance of the thin films. The high coverage of the film is one of necessary conditions for improving the performance. Except for the perovskite film with the highest PCE and full coverage (prepared with 20% of chloride), the morphological structure cannot be reasonably correlated to the performance. Therefore, the crystallinity of perovskite layer should be main parameter contributing to the photovoltaic performance and properties. However, the conventional XRD measurement for all investigated films (Fig. 7) shows that their (110) peak intensities (2θ = 14.3o) have no significant difference and the order of 40% > 20% > 10% > 0% (expressed by chloride ratio), which is not consistent with the PCE result. Noticeably, the GIWAXS result (Fig. 8) can be reasonably correlated to the PCE values. According to 2D GIWAXS patterns, the comparison between the intensities of the out-of-plane (110) diffraction spots of perovskite crystallites (in the Qz direction in Fig. 8) demonstrates the substantial difference in the order of 20% > 40% > 10% > 0%, being consistent with the performance. This present study reveals that the simultaneous GISAXS/GIWAXS analysis can give complete interpretation to the relation between structure and performance. The crystallites with the strong out-of-plane orientation (i.e., the direction normal to the film surface) of (110) plane, shown by 2D GIWAXS (or GIXRD) measurement, is closely related to the high device performance. According to the recent literature^38^, the grain boundary (GB) of crystallites plays a beneficial role in collecting charge carriers efficiently. We speculate that the GB network formed by the dominated crystallites with the out-of-plane orientation (normal to the film surface) has an alignment structure which is more favorable to the transport of charge carrier than that of the crystallites with other orientations. We also correlate the nanomorphology of the films to the crystal orientation. For the perovskite films with internal pore network (prepared with 0% and 10% of chloride), the crystallites oriented to the out-of-plane direction dominate. No crystallites with the in-plane orientation were apparently observed. The dense perovskite films with surface fractal morphology (prepared with 20% and 40% of chloride) have the crystallites with out-of-plane as well as in-plane orientations. The film with over-coalesced grains (40% of chloride) has highly-ordered perovskite structure (as shown by the other spots at high Q region between Qz and Qx directions in Fig. 8).

**Figure 9:**
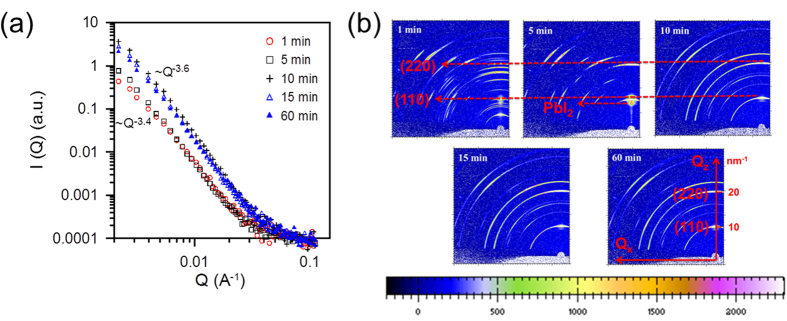
(**a**) GISAXS profiles of the perovskite films prepared with 20% of chloride at 75 °C for 1, 5, 10, 15, 60 min, respectively. (**b**) Two dimensional GIWAXS patterns corresponding to Fig. 9a.

From the mechanistic viewpoint, it is interesting to understand how both the film morphology and crystallization concurrently evolve with annealing time. The GISAXS profiles and 2D GIWAXS patterns of the perovskite films prepared with 20% of chloride at 100 °C for 1, 5, 10, 15, 60 min, respectively, are shown in Fig. 9. The GISAXS profiles annealed for 1 and 5 min have the characteristic of surface fractal morphology with surface fractal dimension of *D*_*s*_ = 2.6 (*α* = 3.4). Their morphology is very similar and may be similar to one before annealing. They seem to be almost stable to the annealing time. The GIWAXS measurement presented here is of much higher resolution compared to the previous measurement because a new 2D high-efficiency detector was adopted here and the experimental technique was updated. The corresponding GIWAXS pattern of the film annealed for 1 min (Fig. 9b) shows the so-called “crystalline precursor structure”, which is consistent with that observed by the other group^23^. This structure partly has the typical perovskite structure with the (110) and (220) spots at *Q* = 10 and 20 nm^−1^. There is also a distinctive set of scattering spots at lower *Q* values (<9 nm^−1^; as indicated in Fig. 9b). The corresponding GIWAXS pattern of the film annealed for 5 min shows the increasing crystallinity of perovskite structure. The peaks at lower *Q* region of the “crystalline precursor structure” disappear and accompany with the formation of the other precursor peak^23^ at *Q* = 11 nm^−1^. However, there additionally appears a signature peak of PbI_2_ at *Q* = 9 nm^−1^, revealing the occurrence of decomposition. This decomposition situations occurring for the short annealing time for the solution-processed perovskite film and at the low substrate temperature for the vacuum-deposited perovskite film (presented in this study) may be due to the oxygen and humidity during handling in the air^19^,^23^. Their common point is the insufficient thermal effect. It can be explained that the incomplete crystallization has more paths to allow the entrance of oxygen and humidity. The GISAXS profiles annealed for 10, 15 and 60 min have the similar characteristic of surface fractal morphology with fractal dimension of *D*_*s*_ = 2.3 (*α* = 3.7). It seems that the morphological evolution of these films get into the other regime (Fig. 9a), different from those annealed for short times (1 & 5 min). Their corresponding GIWAXS patterns and intensities show the stable and complete crystallization. It agrees with the kinetic theory that the development of crystallization tends to be saturation. The PCE values of the devices based on the films annealed for 5, 10 and 15 min are 11, 13.8 and 12.7%, respectively. (J-V curves and EQE spectra are in the Supporting Information, Figure S2). The device based on the film annealed for 5 min has the lower PCE due to the incomplete crystallization. However, The PCE values decreases with the annealing time starting from 10 min (the optimum condition). Because their crystallinities are almost the same, the reduction in PCE may be attributed to the morphological change. According to the GISAXS result (Fig. 9a), the surface fractal dimension gradually increases (the slope of power-law scattering curves decreases) with the annealing time from 10 min to 60 min. It reflects the surface roughness becomes larger; suggesting that smooth interface in the full-coverage perovskite film with complete crystallization is favorable to the performance. This present study demonstrates that GISAXS/GIWAXS characterization can provide the complementary information to the usual XRD and microscopic results.

## Discussion

We demonstrate that the simultaneous GISAXS/GIWAXS can quantitatively investigate the hierarchical structure from the crystal structure and orientation to internal and surface morphology of the CH_3_NH_3_PbI_3−x_Cl_x_ perovskite film. The result is complementary to the conventional microscopic tools. It could successfully provide complete interpretation correlating the multi-length-scale structure to performance for the planar heterojunction perovskite solar cells. Our work reports for the first time that the structural evolution of one-step solution-processed film from the internally fractal pore network to the dense structure with surface fractal morphology by tuning the chloride content. This work provides the insight into the fabrication control. The perovskite crystallites with the orientation of (110) plane normal to the surface are favorable to PCE compared to the other orientated crystallites. The crystallinity is a critical role in the enhancement of PCE. The control of solution-processed CH_3_NH_3_PbI_3−x_Cl_x_ perovskite crystallinity is closely related to the development of full-coverage and internally dense structure. However, the full-coverage and internally dense structure usually do not develop in parallel, which can only be detected by GISAXS method. To achieve the highest PCE, after the full coverage and complete crystallization (suitably measured by 2D GIWAXS detector) attain, the control of surface fractal morphology toward the smoothest surface (i.e., *D*_*s*_ = 2; the corresponding *α* = 4) becomes very important. This rule is suitable to both the vacuum-deposited and solution-deposited CH_3_NH_3_PbI_3−x_Cl_x_ perovskite solar cells. The GISAXS/GIWAXS measurement provides the mechanistic understanding of concurrent evolution of the film morphology and crystallization correlated to the performance.

## Methods

### Device and Thin-film Preparation

The devices were prepared on cleaned ITO substrates by spin coating a 30-nm PEDOT:PSS (Clevios AI 4083) thin film. The mixture of CH_3_NH_3_I, PbI_2_ and PbCl_2_ was dissolved in dimethylformamide (DMF) (total 40 wt% of solute). The mole ratios of 0, 10, 20 and 40% (Cl/(Cl + I); in unit of at.%) samples were (PbI_2_ : PbCl_2_ : CH_3_NH_3_I) = (1 : 0 : 1), (5 : 1 : 8), (5 : 3 : 14) and (0 : 1 : 3), respectively. The solution was stirred at 60 °C for 24 h. The resulting solution was coated on top of the PEDOT:PSS layer by a spin-coating process at 8000 r.p.m for 60 s. The substrates were dried on a hot plate at 100 °C until the perovskite films turned dark brown. For the films prepared with 20% of chloride, the samples were dried on a hot plate at 100 °C for 1, 5, 10, 15 and 60 min. The PCBM layer was then spin-coated onto the perovskite layer using a CB + CF(0.5cc for each solvent) solution of 30 mg/ml. The devices were completed by the vacuum evaporation of Bphen, Ca and Ag. The devices were configured as: Glass substrate/ITO (145 nm)/PEDOT:PSS (30 nm)/perovskite/PCBM (120 nm)/Bphen (6 nm)/Ca (1 nm)/Ag (120 nm). The corresponding bandgap and schematic of our devices are shown in the supporting information (Figure S3). The devices were encapsulated using a UV-cured sealant (Everwide Chemical Co., Epowide EX) and a cover glass under an anhydrous nitrogen atmosphere after fabrication and subsequently characterized in air.

### Thin-Film Characterization

Perovskite thin films for SEM, XRD and absorption measurements were prepared using the same fabrication conditions as the solar cells with layer structures configured as: Glass substrate/ITO (145 nm)/PEDOT:PSS (30 nm)/perovskite (SEM, XRD, absorption). The SEM images were obtained using a Japan Electron Optics Laboratory Co., Ltd. (JEOL) JSM-7000F scanning electron microscope. The X-ray diffraction was performed using a Rigaku TTRAXIII with Cu Kα radiation. The absorption spectra were acquired using a SHIMADZU UV-2600 UV-vis spectrophotometer. The current density versus voltage (J-V) characteristics of the devices were measured using a Keithley SourceMeter 2636A in the dark and under AM 1.5G simulated solar illumination with an intensity of 100 mW/cm^2^ (1 sun, calibrated by a NREL-traceable KG5 filtered silicon reference cell from PV measurements Co.). The device area was determined by the overlap of the ITO and metal electrode with area of 0.12 cm^2^. The EQE spectra were acquired by illuminating a chopped monochromatic light with a continuous-wave bias white light (from halogen lamp, intensity ~100 mW/cm^2^) on the solar cells. The monochromatic light intensities were measured with a NIST-traceable power meter (Ophir). The photocurrent signals were then extracted using the lock-in technique using a current preamplifier (Stanford Research System) followed by a lock-in amplifier (AMETEK).

### Simultaneous GISAXS and GIWAXS Experiment

Simultaneous GISAXS and GIWAXS measurements for the various perovskite layers on Si substrates prepared with the same fabrication conditions were performed at the beam-line 23A of the National Synchrotron Radiation Research Center (NSRRC), Taiwan. In the GISAXS/GIWAXS measurements, the incident angle to each thin film was aligned precisely to 0.2° (slightly lower than the critical angle of Si substrate) for investigating the whole layer and minimizing the background from Si substrate. The scattering intensities were reduced through the standard calibration and background subtraction and are expressed as a function of scattering vector, *Q*, where *Q* = 4π (sin θ)/*λ*, *θ* is half of the total scattering angle, and *λ* is the X-ray wavelength. 2D GISAXS and GIWAXS patterns were collected simultaneously from two 2D detectors located at different positions in the instrumental configuration. The photon energy of beam used is 8 keV. Our previous experiment demonstrate that the difference between the scattering intensity profiles based on the Si and Si/PEDOT:PSS substrates is little. The effect of PEDOT:PSS substrate on the scattering data can be ignored.

## Additional Information

**How to cite this article**: Huang, Y.-C. *et al*. Insight into Evolution, Processing and Performance of Multi-length-scale Structures in Planar Heterojunction Perovskite Solar Cells. *Sci. Rep*. **5**, 13657; doi: 10.1038/srep13657 (2015).

## Supplementary Material

Supplementary Information

## Figures and Tables

**Table 1:** Structural parameters determined by the GISAXS analysis for the hierarchical structure of the one-step solution-processed perovskite films as a function of percentage of chloride.

Chloride percentage (%)	Fractal type	*D*_*s*_	*p*	*R*(nm)	*D*	*ξ*(nm)	*R*_*g*_ (nm)	V_oc_ (V)	J_sc_ (mA /cm^2^)	FF (%)	PCE (%)
0	pore	—	0.58	3.3	1.8	29	28	0.47	15.14	0.44	3.14
10	pore	—	0.54	4.0	2.6	115	115	0.49	18.00	0.56	4.92
20	surface	2.3		—	—	—	—	0.93	21.21	0.70	13.8
40	surface	3.0		—	—	—	—	0.90	20.58	0.61	11.4

The corresponding solar cell device performances are also listed.
