# Supplementary material for: Insight into Evolution, Processing and Performance of Multi-length-scale Structures in Planar Heterojunction Perovskite Solar Cells
Source: Sci Rep. 2015 Sep 4;5:13657. doi: 10.1038/srep13657 (PMC4559897; doi:10.1038/srep13657)
Supplement: Supplementary Information [file srep13657-s1.doc]

Supplementary Information

Insight into Evolution, Processing and Performance of Multi-length-scale Structures in Planar Heterojunction Perovskite Solar Cells

Yu-Ching Huang^1^, Cheng-Si Tsao^1,*^, Yi-Ju Cho^2^, Kuan-Chen Chen^2^, Kai-Ming Chiang^2^, Sheng-Yi Hsiao^2^, Chang-Wen Chen^2^, Chun-Jen Su^3^, U-Ser Jeng^3^, Hao-Wu Lin^2, *^

^1^Institute of Nuclear Energy Research, Longtan, Taoyuan 32546, Taiwan

^2^Department of Materials Science and Engineering, National Tsing Hua University, Hsinchu 30013, Taiwan

^3^National Synchrotron Radiation Research Center, Hsinchu 30077, Taiwan

Corresponding authors: [cstsao@iner.gov.tw](mailto:cstsao@iner.gov.tw); [hwlin@mx.nthu.edu.tw](mailto:hwlin@mx.nthu.edu.tw)


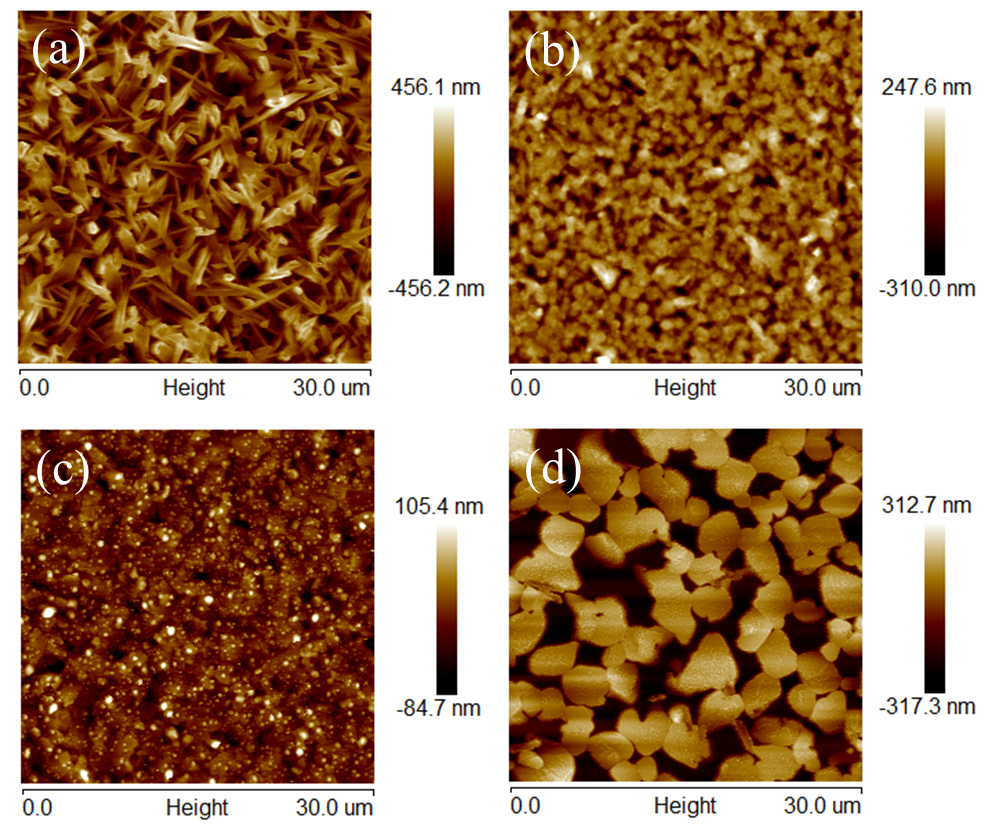


**Figure S1** AFM images of perovskite films prepared with (a) 0, (b) 10, (c) 20 and (d) 40% chloride precursor.


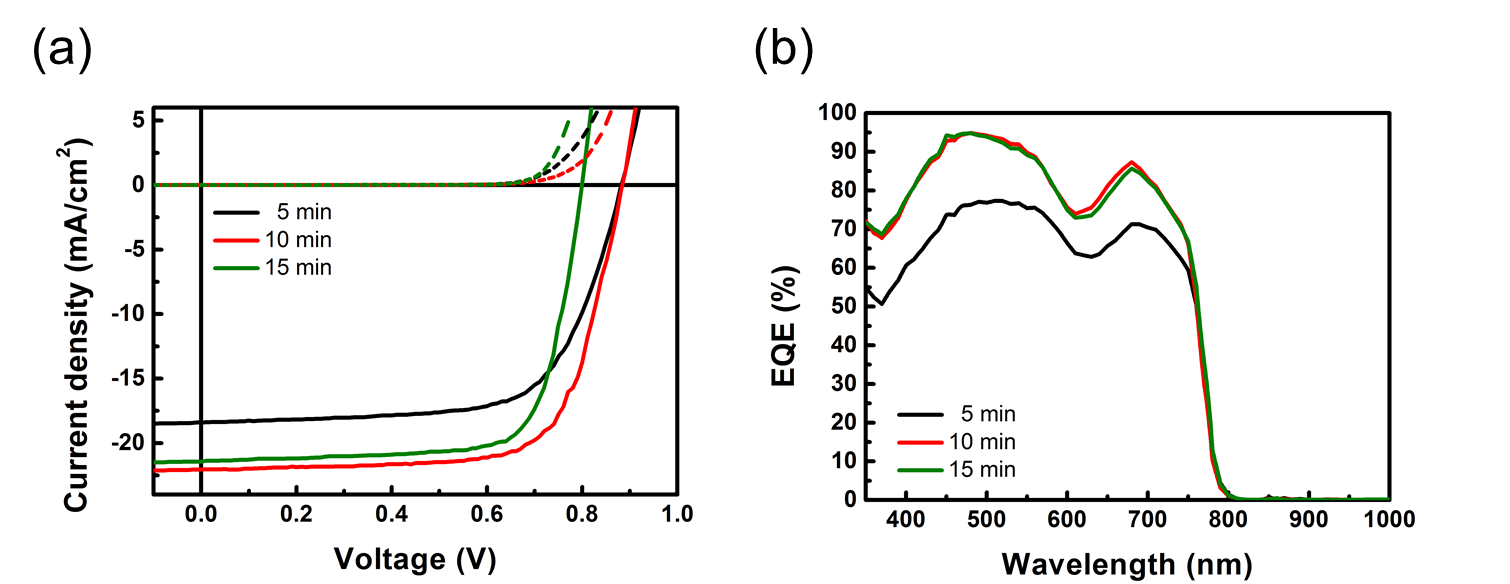


**Figure S2** (a) J-V characterization and (b) EQE spectra of planar heterojunction perovskite solar cells annealed for 5, 10 and15 min.


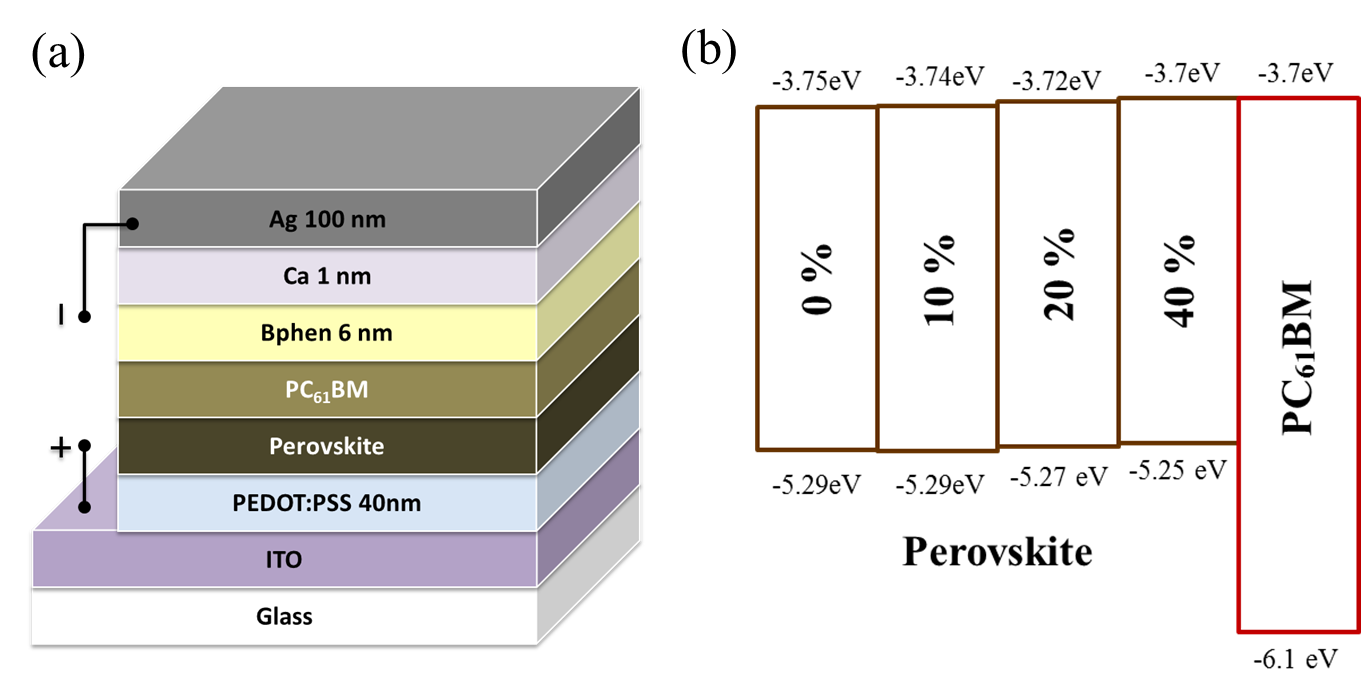


**Figure S3** (a) Schematic of perovskite devices and (b) bandgap of perovskite films prepared with 0, 10, 20 and 40% chloride precursor.
